# The Importance of Genetic and Shared Environmental Factors for the Associations between Job Demands, Control, Support and Burnout

**DOI:** 10.1371/journal.pone.0075387

**Published:** 2013-09-25

**Authors:** Victoria Blom, Lennart Bodin, Gunnar Bergström, Lennart Hallsten, Pia Svedberg

**Affiliations:** 1 Division of Insurance Medicine, Department of Clinical Neuroscience, Karolinska Institutet, Stockholm, Sweden; 2 The Swedish School of Sport and Health Sciences, Stockholm, Sweden; 3 Division of Intervention and Implementation Research, The Institute of Environmental Medicine, Karolinska Institutet, Stockholm, Sweden; University of Hong Kong, Hong Kong

## Abstract

Within occupational health research, one of the most influential models is the Job Demands-Control-Support model. Numerous studies have applied the model to different domains, with both physical and psychological health outcomes, such as burnout. The twin design provides a unique and powerful research methodology for examining the effects of environmental risk factors on burnout while taking familial factors (genetic and shared environment) into account. The aim of the present study was to investigate the impact of familial factors on the associations of burnout with job demands, control and support. A total of 14 516 individuals from the Swedish Twin Registry, who were born between 1959 and 1986, and who participated in the Study of Twin Adults: Genes and Environment (STAGE) by responding to a web-based questionnaire in 2005, were included in the analyses. Of these, there were 5108 individuals in complete same-sex twin pairs. Co-twin control analyses were performed using linear mixed modeling, comparing between-pairs effects and within-pair effects, stratified also by zygosity and sex. The results indicate that familial factors are of importance in the association between support and burnout in both women and men, but not between job demands and burnout. There are also tendencies towards familial factors being involved in the association between control and burnout in men. These results offer increased understanding of the mechanisms involved in the associations between work stress and burnout.

## Introduction

In occupational health research, one of the most influential models is the Job Demands-Control-Support model, JD-C-S [1], [2]. The main assumption is that a combination of high demands and low control (so called high strain jobs) predicts psychological and physical strain, whereas jobs in which both demands and control are high (so-called active jobs) produce well-being, learning and personal growth [3]. Numerous studies have applied the model to different domains with both physical and psychological health outcomes, such as cardiovascular disease, depression, and burnout [3]–[7]. When considering a condition like burnout, which has been shown to be influenced by both genetic and non-genetic factors [8]–[10], a twin study provides a unique and powerful research methodology for examining the effects of environmental risk factors on burnout while taking familial factors (genetic and shared environment) into account. To our knowledge, no previous studies have investigated the impacts of job demands, control and support on burnout using this kind of design.

According to the JD-C-S model, all three aspects are crucial to the development of health problems [3]. Previous findings have shown that there is good evidence for main effects of job demands, control, and support, with the strongest associations for job demands and health outcomes [4]. However, there is only modest support for the moderating influence of control between job demands and health outcomes, and also for the moderating influence of support in three-way interaction [6]. A number of studies have examined the JD-C-S model in the context of burnout, and have found main effects of job demands, control, and support [11]–[13].

Burnout is regarded as a work-related stress syndrome, often defined on three dimensions: exhaustion, feelings of cynicism and disconnectedness from the job, and a sense of ineffectiveness at work [14]. Exhaustion has been thought to be the central, basic stress dimension among these three components, and is characterized by an overwhelming sense of overstrain and being worn down [14]. Even though burnout is conceptually close to depression, studies have shown that they are indeed separate syndromes, with separate etiologies and outcomes, and different physiological expressions [15], . In the present study, burnout is measured using the Pines Burnout Measure (Pines BM) [17], which is a context-free measure of burnout symptoms that can be applied to any group, such as students or unemployed, and to people in employment and on sick-leave. Composite scores on the Pines BM correlate substantially with the exhaustion dimension in the Maslach Burnout Inventory (MBI) [18], and MBI and Pines BM have been found to distinguish between burned-out and non-burned-out individuals equally well [19]. Across studies, burnout has been found to be significantly more common in women than men, and among younger rather than older people [14].

The co-twin control design [20]–[22] compares how differences in exposures (such as work stress) within twin pairs contribute to differences within the twin pairs on an outcome (such as burnout). Monozygotic, identical (MZ) twins share all of their genetic material, whereas dizygotic, fraternal (DZ) twins share, on average, 50% of the segregating genes. Differences between MZ twins are therefore likely to reflect environmental effects. Moreover, DZ twins are the perfect comparison group for MZ twins since both MZ and DZ twins are most likely influenced by similar early life-environment factors, such as socioeconomic status or upbringing, which can affect later life outcomes, such as occupation. In the present study, co-twin control design of same-sex twin pairs enables investigation of the impact of work stress on burnout, while controlling for familial factors, age and sex.

Previous studies not based on twin data have found that adversities in adolescence within families, such as residential mobility and crowding, parental loss, and parental unemployment, can affect self-rated job strain in adult age, in particular in women [23]. Furthermore, twin studies have found a genetic component in burnout [8], [9], and also in coping with professional demands [24] and social support [25]. Genetic factors have also been found to contribute to the associations between stressful life events and depression [26], and between unemployment and anxious depression [27]. On these grounds, there are reasons to believe that genetic and shared environmental factors can influence the associations between job demands, control, support and burnout.

The aim of the present study was to investigate the impact of familial factors on the association between work stress and burnout, where work stress was assessed using the Job Demands-Control-Support model (JD-C-S).

## Method

### Ethics statement

This study was based on the twins who participated in the STAGE study administered by the Swedish Twin Registry (STR) in 2005. An application was sent to the STR, who approved the request to use already collected data (2/17/2009). Informed consent was collected for the STAGE study, as too was concomitant ethical evaluation and approval (03-224, 5/5/2003). Ethical vetting was performed by the regional ethical review boards. Pursuant to standards in Sweden, the project was evaluated and approved by the Regional Ethical Review Board of Karolinska Institutet, Stockholm, Sweden (2009/2053-31/5, date 18/2/2010).

### Participants

The source population consisted of 25 378 MZ and DZ twin individuals from the Swedish Twin Registry (STR), who were born between 1959 and 1985, and who participated in the Study of Twin Adults: Genes and Environment (STAGE) by responding to a web-based questionnaire in 2005 [28]. The source population represents various groups, such as students, people employed in various sectors and professions, and persons on sick-leave. Since the aim of the study was to investigate the impact of work stress on burnout, responses were excluded from individuals who, at the time of the survey, were not employed. Thus, a total of 14 516 individuals were included in the study group, where of 53% worked in the private sector, 20.5% in municipalities, 9% in the public sector, and 7% in county councils; 6.5% were self-employed, and 3% were employed in other sectors. Further, 56% were women and, at the time of the study, all participants were between 20 and 46 years-old. Forty-two percent indicated they had attended Swedish high school, and 42% had a university level degree. In the co-twin control analyses, 5108 individuals (2554 same-sex twin pairs) with complete information on Pines BM, control, support and job demands were included. Of these, 2894 were MZ and 2214 DZ twins. See Table 1 for details of the numbers of participants and exclusions.

**Table 1:** Numbers of twins in the source population of working twins and formation of study group for different analyses.

Number of twins	Single twin/Individuals in complete pairs	Exclusions
16 412	7648/8764	293 Unknown Zygosity
		1603 Missing value for Burnout
14 516	7280/7236	6689 No possibility to form within-twin mean values and differences for Job demands, Control and Support
7827^1^	627/7200	627 No possibility to form within-twin mean value and difference for Burnout
7200	0/7200	2092 Opposite sex twins

5108 twin individuals in complete pairs available for co-twin analysis: 2894 MZ, 2214 DZ same sex.

1 In the descriptive analyses 7378 twins were included, randomly drawn from each twin pair in order to account for dependence between twin pairs.

The zygosity of the same-sex twin pairs was determined in the STAGE study on the basis of questions about childhood resemblance. When validated against serological and micro-satellite markers, this method is about 98% accurate [29].

### Measures

Burnout was measured as a state of exhaustion using three items from the Pines BM [17], expressed as “Feeling depressed”, “Being emotionally exhausted”, and “Feeling run down”. Responses were given on a seven-point Likert scale ranging from 1 =  “do not agree” to 7 =  “agree entirely”, with a higher score indicating a higher level of burnout. Further, the three items in the Pines BM included in STAGE, and hence available for the present study, were chosen as they were found to correlate strongly (r = 0.90) with the full 21-item Pines BM [30]. In the present study, Cronbach's alpha for the three-item scale was .89.

The Swedish translation [31] of Karasek and Theorell's [2] measure was used to assess job demands, control and support, as expressed, for example, by “Does your job require too great a work effort?” (job demands), “Do you have the possibility to decide for yourself how to carry out your work?” (control), and “There is good collegiality at work” (support). Responses were given on a four-point Likert scale. Scores on the items were reversed, except in two cases (“Do you have sufficient time for all your work tasks?”, and “Does your work require doing the same tasks over and over again?”), so as to refer to 1 =  “do not agree” to 4 =  “agree entirely”. All measures are thus interpreted in terms of a higher score indicating greater perceived demands, control and support. The control dimensions, skill discretion and decision authority, were used as two separate measures, and also combined into one measure of control.

### Statistical analyses

First, descriptive analyses were performed on a random sample of twins drawn from each twin pair in the cohort sample in order to adjust for dependence between the twins. Multivariate analysis of variance (MANOVA) was used to establish differences between women and men with regard to job demands, control and support, and burnout, and Pearson correlations for associations between the included variables.

The following analyses were performed in complete same-sex twin pairs in accordance with recognized procedure in co-twin control design [20], [21]. By excluding opposite sex twins it is possible to control for sex, age, genetics and shared environment [20], [21]. However, analyses including opposite sex twins were also performed in order to compare the results of including and excluding these twins. First, two models were compared according to Carlin's recommendations [20] by calculating the main effects of job demands, control and support on burnout, and also the moderating effects of control and support between job demands and burnout. This was performed without acknowledgement of co-twin scores (Model 1), making the results comparable to those from a non-twin sample. However, as twins are not independent of each other, a linear mixed model with a correction for this dependence was employed. In Model 2, the effects of job demands, control and support on burnout between and within the twin pairs were analyzed. In these analyses, sex and zygosity were included as confounders. At the next step, the co-twin control analyses based on Model 2 were stratified by sex and zygosity, so as to further analyze the between-pairs and within-pair effects. Stratification on sex was performed, since both burnout and JD-C-S have been found to differ between men and women [6], [14].

The regression models for the effects of burnout on the JD-C-S variables were estimated using procedures for linear mixed modeling based on maximum likelihood estimation, with allowance for dependence within the twin pairs. The goodness of fit of the different model specifications was tested by likelihood ratio tests, supplemented by Akaike's information criterion. A significance test for differences between MZ and DZ twins was performed using Wald's method.

The between-pairs variable was calculated as the mean levels of job demands, control and support of the twin pairs, and the within-pair variable as each twin's difference from the pair mean. The within-pair effect was matched on all shared environmental and genetic factors (100% for MZ pairs, and on average 50% for DZ pairs). The interpretation of the co-twin analyses involved comparisons of the between-pairs and within-pair effects to detect the presence of familial factors, and also comparisons between the MZ and DZ pairs to establish whether shared environmental factors or genetic factors accounted for the effects. A significant within-pair effect represents an association that is not confounded by factors shared by the two twins in a pair [20], [21]. However, if the between-pairs effect differs significantly from the within-pair effect, factors common to the twins in a pair are involved in explaining the association. If there is no difference between the between-pairs and within-pair effects, Model 1 can be used to account for the effects. Moreover, if the between-pairs and within-pair effects differ similarly in MZ and DZ twins, shared environmental factors can be regarded as being more involved than genetic factors [20], [32], [33].

All variables were treated as continuous variables to make use of the full information available about them. The IBM SPSS 20 and Stata 12.0 packages were used for the statistical analyses. The study was approved by the Regional Ethical Review Board in Stockholm, Sweden.

## Results

Descriptive statistics for the whole sample, including twins randomly drawn from each twin pair in order to account for dependence between twins in the same pair, are shown in Table 2. Pearson correlation coefficients showed that job demands and support were moderately associated with burnout. The MANOVA showed small but significant differences between men and women for burnout (F = 390.38, p<.001, η^2^ = .05), support (F = 11.72, p<.001, η^2^ = .01) and control (F = 93.94, p<.001, η^2^ = .01), but not for job demands (F = .61, p>.05, η^2^ = .00).

**Table 2:** Mean values (standard deviations) and correlations in a sample of Swedish twins, randomly drawn from each twin pair (n = 7378).

	Burnout	Job demands	Support
**Burnout**			
Women			
2.71 A (1.30)			
n = 3769			
Men			
2.15 A (1.12)			
n = 3609			
**Job demands**			
Women	.23**		
2.72(.52)			
n = 3769			
Men	.20**		
2.73 (.50)			
n = 3609			
**Support**			
Women	−.27**	−.27**	
3.34 A (.50)			
n = 3769			
Men	−.23**	−.25**	
3.38 A (.47)			
n = 3609			
**Control**			
Women	−.09*	.13*	.22**
2.99 A (.56)			
n = 3769			
Men	−.07*	.12**	.22**
3.12 A (.56)			
n = 3609			

**<.001,

*<.05,

A = significant mean difference between men and women.

Model testing with likelihood ratio tests, and also Akaike's information criterion, was performed, showing that Model 2 had significantly better fit than Model 1, indicating that familial factors are of importance in the data (Table 3). It was also found that, for Model 2, the effects of job demands, control and support on burnout did not interact with either sex or zygosity, at least in the sense that no interaction reached statistical significance. Hence, the subsequent analyses focused on comparing Model 1 and Model 2, with sex and zygosity included as covariates and as stratification variables, but with no interaction terms.

**Table 3:** Likelihood ratio tests of different specifications of linear mixed models for analyses of the relations between burnout and job demands, control and support.

Model with the smallest number of parameters	Model with the largest number of parameters	Likelihood ratio p-value^1^
Model (1)	Model (2)	**26.17**
		**p<0.0001**
Model (2)	Model (2) + interactions sex*JD-C-S^2^	8.31
		p = 0.22
Model (2)	Model (2) + interactions zygosity*JD-C-S	2.72
		p = 0.84
Model (2)	Model (2) + interactions sex*JD-C-S and zygostity*JD-C-S	11.12
		p = 0.43
Model (2)	Model (2) +3-way interactions sex*zygosity*JD-C-S	20.45
		p = 0.37
Model (2)	Model (2) + interaction sex*zygosity	0.90
		p = 0.34

Two different models, Model (1) and Model (2), for twin analysis according to Carlin et al. [20] (5108 individuals in complete same-sex twin pairs).

1 p<0.05 indicates improved model fit for the model with the largest number of parameters. Statistical significances are shown in bold.

2 JD-C-S is shorthand for Job Demands, Control and Support.

The regression parameters from the co-twin analyses, separately for Model 1 and for Model 2, following Carlin [20], are reported in Table 4. Model 1, comparable to a non-twin sample, showed that there were significant effects of job demands, control and support on burnout, with the strongest predictors being perceived support (β = −.47, p<.001) and perceived demands (β = .42, p<.001). When support increased one point on a four-point scale, the level of burnout level decreased by .47 on a seven-point scale. Control showed a weaker association (β = −.13, p<.001). There were no significant interaction effects of job demands and control on burnout (β = .01, p>.05), or of job demands, control and support on burnout (β = −.01, p>.05). Moreover, the same pattern of results, with main effects but no interaction effects, emerged when the two dimensions of the control variable were used, i.e., skill discretion and decision authority. Since these control dimensions showed similar results to the composite control variable, neither of these dimensions, nor the interaction variables, are shown in the table, or were included in the subsequent analyses. In addition, analyses reported in Table 5, including also opposite sex twins in the model and expanding the data to 7200 subjects, showed results in close agreement with those reported for the strict co-twin analyses in Table 4. A likelihood ratio test of the difference between same- and opposite sex twin pairs with respect to the model of Table 5 gave p = .94.

**Table 4:** Linear mixed model analyses of the associations between burnout and job demands, control and support in two different models for twin analysis, Carlin et al. [20].

	Model (1)		Model (2)					
	B_c_	CI (95%)	B_B_	CI (95%)	B_w_	CI (95%)	B_B_−B_w_	CI (95%)
**Job demands**	.42**	.35–.49	.43**	.33–.53	.40**	.30–.49	.03^ns^	−.10–.17
**Support**	−.47**	−.55–−.40	−.65**	−.75–−.54	−.32**	−.42–−.22	−.33**	−.47–−.18
**Control**	−.13**	−.20–−.07	−.13*	−.22–−.04	−.14*	−.23–−.05	.01^ns^	−.12–.13

The models include sex and zygosity as additional covariates (5108 individuals in complete same-sex twin pairs).

**p<.001,

*p<.05,

ns not significant.

**Table 5:** Linear mixed model analyses of the associations between burnout and job demands, control and support in two different models for twin analysis, Carlin et al. [20] including both same- and opposite-sex twins.

	Model (1)		Model (2)					
	B_c_	CI (95%)	B_B_	CI (95%)	B_w_	CI (95%)	B_B_−B_w_	CI (95%)
**Job demands**	.42**	.37–.48	.46**	.38–.54	.39**	.31–.46	.07^ns^	−.04–.18
**Support**	−.49**	−.55–−.43	−.64**	−.72–−.55	−.35**	−.43–−.26	−.29**	−.41–−.17
**Control**	−.10**	−.15–−.04	−.07*	−.14–−.00	−.13*	−.20–−.05	.06^ns^	−.04–.16

The models include zygosity as additional covariate (7200 individuals in complete same- and opposite-sex twin pairs).

**p<.001,

*p<.05,

ns not significant.

Since the between-pairs and within-pair effects differed significantly with regard to support (B_B_−B_W_ = −.33, 95% CI −.47–.18), Model 2, on this criterion, was regarded as informative in terms of understanding the influence of familial factors [20]. Thus, to gain further insight into the factors involved in Model 2, the subsequent analyses focused on that model (Table 6). Even though interaction terms with sex were not significant, these co-twin control analyses were stratified by sex and zygosity, since previous research has found differences between men and women with regard to burnout and JD-C-S [4], [14].

**Table 6:** Linear mixed model analyses, stratified by sex and zygosity, of the associations between burnout and job demands, control and support, with separation between between-pairs and within-pair effects; Model (2) from Carlin et al. [20] (5108 individuals in complete same-sex twin pairs).

		B_B_	CI (95%)	B_w_	CI (95%)	B_B_−B_w_	CI (95%)
**Job demands**	**n**						
MZ, Men	1272	.37**	.19–.55	.38**	.21–.56	−.01^ns^	−.26–.24
DZ, Men	936	.32*	.12–.52	.49**	.28–.70	−.17^ns^	−.46–.12
MZ, Women	1622	.46**	.28–.64	.35**	.17–.53	.11^ns^	−.14–.37
DZ, Women	1278	.54**	.34–.75	.39**	.19–.60	.15^ns^	−.15–.44
**Support**							
MZ, Men	1272	−.70**	−.89–−.51	−.18*	−.36–−.00	−.52**	−.78–−26
DZ, Men	936	−.49**	−.69–−.28	−.43**	−.63–.23	−.06^ns^	−.35–.23
MZ, Women	1622	−.68**	−.88–−.48	−.34**	−.53–−.16	−.33*	−.61–−.06
DZ, Women	1278	−.68**	−.88–−.48	−.33*	−.55–−.11	−.35*	−.65–−05
**Control**							
MZ, Men	1272	.11^ns^	−.05–.26	−.25*	−.41–−.10	.36**	.14–.58
DZ, Men	936	−.05^ns^	−.22–.11	−.08^ns^	−.26–.10	.02^ns^	−.22–.27
MZ, Women	1622	−.16*	−.32–−.01	−.03 ns	−.20–.14	−.13^ns^	−.36–.10
DZ, Women	1278	−.11^ns^	−.30–.08	−.20*	−.39–−.01	.09^ns^	−.18–.36

**p<.001,

*p<.05,

ns not significant.

The co-twin control analyses stratified by sex and zygosity (Table 6) showed no significant differences between between-pairs and within-pair effects for job demands and burnout, and for control and burnout except in the case of MZ men. For support, on the other hand, there were significant differences for all subgroups except for DZ men. Based on observations from the model testing (shown in Tables 3–6), we suggest a parsimonious model that is a mixture of Model 1 and Model 2, with support differentiated into between-pairs and within-pair effects, but with job demands and control not differentiated. The parsimonious model showed a better fit than Model 1 (likelihood ratio test, p<0.0001), and an almost as good a fit as Model 2 (likelihood ratio test, p = 0.30), thus supporting our suggestion that familial confounding is involved in the association between burnout and support, but not between burnout and job demands or control.

Wald's test, taking into account both zygosity and between-pairs and within-pair effects, showed that the between-pairs and within-pair effects differed similarly in DZ and MZ twins for women in all the associations. However, for men the coefficients differed between MZ and DZ twins for both support and control (p<.05).

## Discussion

The aim of the study was to investigate the impact of work stressors, as assessed by the JD-C-S model, on burnout, while controlling for familial influences. There is a vast amount of research into the JD-C-S model in relation to health outcomes, but as far as we know, there are no studies that have included adjustments for genetic and shared environmental factors.

The main and interaction effects estimated without account taken of the co-twin scores were in line with previous research [4]–[6], [34]. Moreover, as found in other studies [4], [6], women scored somewhat lower on support and control than men, but not significantly differently from men on job demands. This suggests that the twin sample in the present study does not differ from other populations, and thereby strengthens the validity of the results.

The co-twin control results suggest that familial factors are involved in the association between support and burnout for both women and men. This is in line with previous research showing a genetic component in social support [25], and also studies of familial confounding in the associations between environmental stressors and health outcomes, such as depression [26]. The differences between within-pair and between-pairs effects were similar in MZ and DZ female twins, suggesting that shared environmental factors may contribute to the association between social support and burnout to a greater extent than genetics in women. The shared environmental factors might, for example, be attitudes within families, socioeconomic status, and psychosocial factors shared within the families. However, for men, it may be genetic factors rather than shared environmental factors that are most heavily involved in support and burnout. Nevertheless, the validity of interpretations that distinguish between genetic and shared environmental influences has been disputed [20], [35], and the results need to be further elaborated in future research.

With regard to job demands and burnout, familial factors do not seem to be involved among either women or men. This points to a rather direct association between job demands and burnout, which is not affected by genetic or shared environmental factors. There are possibly other factors, not shared by twins, that might affect the association, such as skill level and coping strategies, which, as a third variable, can affect both job demands and burnout. Even so, these are interesting results that draw attention to the benefits for employers of reducing employees' job demands per se in order to reduce burnout and possibly other stress-related ill-health.

The results suggesting that familial factors are involved in the association between control and burnout for MZ men should be interpreted with caution, since the association is weak. This may partly be due to the fact that control includes both skill discretion and decision authority [4], [23], [36]. Moreover, as the present study is based on a fairly young cohort, control can be perceived as another kind of job demand rather than as positive autonomy, in particular among women, as discussed by for instance Westerlund and colleagues [23], which could make the control measure a biased measure in the present study.

Thus, the association between job demands and burnout does not seem to be confounded by familial factors, although such factors seem to be involved in the association between support and burnout, albeit with significant within-pair effects indicating that familial factors do not fully carry the effect of work stress on burnout. Previous studies of the association between JD-C-S and burnout, without adjustment for familial influences, are therefore indeed valid, and are suitable as benchmarks for implementation efforts in the workplace. However, since familial factors seem to be of significant importance with regard to support, there are reasons to take other than work-related aspects into account when investigating the mechanisms underlying the link between JD-C-S and burnout.

A limitation to the present study is that burnout was measured using a short version of Pines BM, which essentially measures exhaustion; in future twin studies, it would be of interest to use, for example, the multi-dimensional Maslach Burnout Inventory. However, exhaustion has been found to be the most central aspect of burnout, and high exhaustion seems to be a more or less necessary link to the potential development of cynicism [37]. Another limitation is that individuals over the age of 46 were not included in the study population, which could reduce generalizability to older age groups. Possibly, familial factors show a weaker effect of work stress on burnout in these groups, since genes are found to have a less profound impact at older ages [38]. The main strength of the study is that, through its use of the unique natural experiment provided by a twin sample, it allows for control of genetics and shared environment, which opens many opportunities to investigate the mechanisms underlying the risk factors for certain health outcomes. However, the results would benefit from being further elaborated, and it is recommended that longitudinal research is performed in order to test for the directions of effects.

To conclude, the main results of the present study are that familial factors are important in the association between support and burnout in both women and men, but not in the association between job demands and burnout. There are also tendencies for familial factors to be involved in the link between control and burnout among men. Moreover, genetic factors may be more involved than family environment among men, while the opposite applies to women. This is new knowledge within the occupational health arena that offers further understanding of the mechanisms involved in the association of work stress with burnout.
